# Conscientious Objection: A Talmudic Paradigm Shift

**DOI:** 10.1007/s10943-019-00979-4

**Published:** 2020-01-10

**Authors:** Rabbi Jason Weiner

**Affiliations:** Cedars-Sinai Medical Center, Los Angeles, CA USA

**Keywords:** Conscientious objection, Jewish medical ethics, Talmud, Conscientious refusal, Abortion, Physician aid-in-dying, Conscience, Judaism

## Abstract

Conscientious objection remains a very heated topic with strong opinions arguing for and against its utilization in contemporary health care. This paper summarizes and analyzes various arguments in the bioethical literature, favoring and opposing conscientious objection, as well as some of the proposed solutions and compromises. I then present a paradigm shifting compromise approach that arises out of very recent Jewish bioethical thought that refocuses the discussion and can minimize the frequency with which conscientious objection is required.

## Introduction

Most medical professionals are guided primarily by their own deeply held morals and values, not what their professional organizations require of them (Davis 2012; Curlin et al. 2008; Curlin 2007). Some healthcare providers do not feel comfortable participating in certain interventions that go against their personal core values, and as medicine achieves the ability to accomplish more and more, interventions become available that present more challenges. Refusal to participate in these interventions is known as “conscientious objection,” which has been defined as “the rejection of some action by a provider, primarily because the action would violate some deeply held moral or ethical value about right and wrong” (Odell et al. 2014).

The issue of conscientious objection arose in modern medicine when some physicians refused to comply with abusive forms of medicine forced onto a population by totalitarian regimes, as was the case in Nazi Germany (Beal and Capiello 2008). It still comes up in contemporary society regarding healthcare professionals, such as doctors, nurses, and pharmacist’s participation in numerous procedures, such as euthanasia, physician aid-in-dying, contraception, and some reproductive technologies. Following the 1973 Roe v. Wade abortion decision,^1^ the United States Congress passed “conscience clause” legislation, known as the “Church Amendment,” which protected an individual’s right of refusal to take part in abortion and sterilization (42 *U.S.C.* 300a-7).^2^ This amendment prohibited courts and government agencies from requiring individuals or facilities to perform abortions or sterilizations if they had moral objections and protected individuals from employer discrimination if they were unwilling to perform abortions or sterilizations (Pope 2010).

Such legislation has not been without controversy, and several states, as well as many countries, have taken extremely divergent approaches (Munthe 2017; Pope 2010). The debate surrounding the appropriateness of healthcare providers engaging in conscientious objection has been intense and often polarizing. This paper will summarize various arguments in the bioethical literature, favoring and opposing conscientious objection, some proposed solutions, and then present a paradigm shifting compromise approach that arises out of very recent Jewish bioethical thought.

## Arguments in Favor of Conscientious Objection

While some argue that conscientious objection is a matter of freedom to practice one’s religion (Sulmasy 2017), most feel that the foundation for exercising this right is even more fundamental (Weinstock 2014). Many contend that a multicultural and tolerant society should simply not be attempting to impose ethical beliefs on others, but rather strive to accept various moral viewpoints. They thus see respect for conscientious objection as a form of tolerance for “moral diversity” (Wicclair 2000; Wear et al. 1994; Engelhardt 1986). Similarly, allowing conscientious objection is a type of ethical modesty or humility, because it recognizes that one might be mistaken about what they think is the correct moral belief, and should thus not be dogmatic of differing viewpoints (Sulmasy 2008). To obligate one to go against their religious or ethical convictions is also regarded as a form of discrimination and a violation of ethical and human rights (Dickens and Cook 2000). Some even see conscientious objection as being integral to a functioning democracy. They suggest that just as people should have the essential right to object to being drafted into military service, they should also have the right to refuse to engage in behaviors they see as immoral (Cantor 2004).

However, what a professional can object to is limited, and some things cannot be tolerated, such as unnecessarily violating patient confidentiality or engaging in discrimination, based on race, sexual orientation, etc. (Eberl 2019). Some thus claim that it is the principle of respect for autonomy that is the strongest basis for both a patient’s right to refuse treatments and a clinician’s right not to engage in them (Brownlee 2012). Respect for individual autonomy implies that professionals should not have to abandon their morals in order to get a job, or leave their job if new interventions become required of them, and that individuals should be allowed to select which procedures they are comfortable participating in, as part of a right to free choice of employment (Myskja and Magelssen 2018). Just as reproductive rights and abortion are often seen as matters of personal choice, so should a professional have a choice *not* to participate (Cantor 2004).

Beyond autonomy arguments, many thinkers frame the importance of conscientious objection as most fundamentally respecting the need of individuals to maintain their “moral integrity” (Wicclair 2019; Benjamin 2004; Childress 1979, 1997; Blustein 1993). This formulation allows healthcare providers to see themselves not only as technicians (Childress and Siegler 1984), like auto mechanics, but recognizes that someone who engages in procedures that go against their conscience and violates their own morality will feel that they are not being true to themselves (Norberg et al. 2014). Such moral distress can cause a loss of self-respect (Wicclair 2019) and may lead to burnout, fatigue, and emotional exhaustion (Lachman 2014; Meltzer and Huckabay 2004). In addition to such possible negative implications, some make the claim that a professional only becomes and remains a responsible moral agent through a gradual process of observation, reflection, and practice, which must be protected so that it can develop (Neal and Fovargue 2019).

From this perspective, protecting the moral integrity of healthcare professionals can benefit our entire society, since many people would want to have their own morality and integrity respected, and everyone benefits from having medical professionals who are trustworthy, honest, and moral public servants. Such behavior would not be promoted if professionals were asked to forsake their ethics in some situations yet apply them to patient welfare in other areas (Hepler 2005). Moreover, allowing students and residents conscience-based exemptions might increase their ethical sensitivity.^3^ Protection of moral integrity and a healthcare professional’s right to refuse to participate thus promotes the common good, as it enables society to benefit from professionals who are encouraged to practice with these virtues (Magelssen 2011). Some also make the case that allowing practitioners to set limits on the interventions that they are morally comfortable performing allows healthcare professionals to practice their vocation with individual integrity and thus earn their patients trust (Neal and Fovargue 2019). While some will argue that the public good is ultimately not served by restricting access to health care, if other providers are willing to offer such services without excessive burden to the patient, a pluralistic society is better served by respecting the interests of as many individuals’ rights as possible—both those in search of these services and those who do not want to engage in them (Wicclair 2008).^4^

## Arguments Against Conscientious Objection

There is also principled opposition to the idea that medical professionals should have a right to conscientious objection. One example is the “incompatibility thesis,” which maintains that medical professionals voluntarily join a profession that is expected to serve society by providing essential services, all of which are legal and professionally accepted, and refusing to do so is incompatible with their professional responsibilities (Neal and Fovargue 2019; Schuklenk and Smalling 2017; Wicclair 2011). From this perspective, medical professionals have an obligation to use their skills to serve their patients, placing the interests of their patients above their own (often referred to as “patient-centered care”). Furthermore, since they have freely chosen this field—unlike a military draft—some would argue that they must adopt its obligations (Stahl and Emanuel 2017).^5^ Those who take this approach argue that individuals should not go into a line of work in which they are conflicted and unable to carry out all of its professional expectations (Cantor 2009; Savulescu 2006),^6^ and particularly not a specialty that includes procedures one is unwilling to engage in (Stahl and Emanuel 2017).

Accordingly, although everyone is entitled to have their own values in their private lives, some see medical professionals as public servants who should thus minimize the extent to which their own values impact communal interests (Stahl and Emanuel 2017; Savulescu 2006). Moreover, allowing conscientious objection places burdens on patients and compromises their care by lowering the quality of health care (Ibid.). This is because it can render health care inconsistent, since treatment can become dependent upon the values of a given doctor, and inefficient, since people must search for doctors who are willing to provide the services they need (Ibid.). Limiting patient choice may be less detrimental to patients who have access to many options, but it disproportionately impacts the poor, those living in rural areas and those with limited mobility (Wicclair 2019; Cantor 2004).^7^ Conscientious objection thus raises social justice concerns since it can severely negatively impact the health of the most vulnerable, which is unfair, unjust, and unnecessarily exposes them to risk. Societal needs should therefore override individual practitioners’ personal, religious or moral needs. As practitioners gain more and more opportunities to refuse treatments, they argue, the potential for various forms of discrimination and misuse of this right will increase (Savulescu 2006; Cantor 2004).^8^

Many thinkers reject the conflation of conscientious objection with morality and its contribution to a just society. They argue that both negative duties and positive duties and claims of conscience exist. Therefore, just as many speak of *refusal* to engage in a given procedure as conscientious objection, those who believe in *providing* such services often do so out of a commitment to core ethical beliefs, which can frame their practice as “conscientious provision” (Harris 2012). From this perspective, moral integrity can also be injured by *not* allowing an action required by one’s core commitments, just as performing an action that contradicts those beliefs can cause moral injury for others. Focus on protecting conscientious objection can thus stigmatize those in need of services as well as those who provide them, and can therefore undermine virtue and commitment to moral ideals in society (ibid.).

## Solutions

While these different perspectives may seem to make much of this issue unresolvable, there are some compromise, or middle-ground, approaches that have been offered. General consensus is that some limits on conscientious objection may be appropriate (Neal and Fovargue 2019). One suggestion is that interventions for which conscientious objection may be requested should be limited, only allowing those with direct participation to object, as well as requiring objectors to satisfy certain tests of genuineness, reasonableness, or rationality before being permitted to refuse involvement (Ibid.; Eberl 2019).

Broader support exists for requiring steps to ensure that conscientious objection has minimal negative impact on the quality, efficiency, and equitability of health care. To achieve that goal, many feel that healthcare providers should at the very least always:

- offer emergency lifesaving services when no alternative is available (Nelson 2018)
- clearly inform patients and employers well in advance of what services they would not be willing to perform (Lyerly 2012; Beal 2008)
- never obstruct access to health care and ideally provide other options to receive those services, including respectfully providing information, options or making a timely referral (Ibid.).

Many conscientious objectors are unwilling to provide referrals for services that they morally object to (Trigg 2017), though some have found ways of making indirect referrals (Norberg et al. 2014). Some argue that when making referrals for procedures one is unwilling to perform, providers should pay for the increased costs their patients may incur (Ancell and Sinnot-Armstrong 2017), and others suggest that those unwilling to provide these minimal accommodations should be disciplined (Savulescu 2006).

## Jewish Approach

Rabbinic thinkers have dealt with this question, and new approaches have been offered in recent years. While much of the above comes out of Christian—especially Catholic—opposition to engage in certain medical interventions, traditional Jewish law has many of the same concerns, but offers a unique perspective that frames this conscientious objection differently and can potentially provide a very helpful compromise approach for conflicted practitioners.

The way conscientious objection becomes categorized in Jewish law takes the focus away from practitioner’s own moral commitments and focuses on the results of one’s actions. Jewish law forbids an observant Jew from transgressing the Torah and also includes some rabbinic prohibitions that serve as safeguards but are not required as strictly as biblical prohibitions. The question Jewish law asks is how concerned must one who observes Jewish law be when other people seek their support in doing something that violates Jewish law, and what biblical verse or category of rabbinic law does this fall under? Rabbinic authorities have placed the prohibition against taking part in another person’s forbidden action under the category of the biblical prohibition against placing a stumbling block before the blind (Leviticus 19:14). By categorizing conscientious objection this way, Jewish law can permit practitioners to do things like provide referrals, engage in ancillary support such as anesthesiology, etc., for most interventions, including those which biblical law prohibits, if the action is going to happen anyways.

Here’s how: traditional rabbinic commentaries have understood this verse to be not just about actually tripping a blind person, but also to contain two fundamental prohibitions that take “blindness” more metaphorically:

1. This verse prohibits giving bad advice to a person who is “blind” in a certain matter or to the consequences of that advice (i.e., informational blindness).^9^
2. The verse also prohibits giving someone the means, causing or facilitating an opportunity for them to stumble morally by transgressing the Torah.^10^

The Talmud and most medieval commentators focus primarily on this second, more expansive prohibition, which leads to much concern in Jewish law about the ramifications of one’s actions and hence the results-based categorization of conscientious objection in Jewish legal sources. The classic example in the Talmud is a prohibition against giving a cup of wine to a person who has vowed not to drink wine (a “*Nazir*”), since giving them the cup can enable them to transgress their vow by drinking from it.^11^

While enabling sin is prohibited by this verse, this prohibition also has some crucial limits that are relevant for medical professionals. Most significantly, the Talmud rules that this commandment is violated only when the person extending the forbidden item and the person receiving it are located on two opposite sides of a river, which completely separates them, such that the person who is “morally stumbling” would be physically unable to access the forbidden item without the assistance of the other person. Extending the forbidden item across the river places a “stumbling block” by creating the opportunity to sin and is thus prohibited by this verse.^12^ However, one would not violate this verse by passing a cup to the person if both of them are on the same side of the river, meaning that the transgressor could have engaged in that prohibited action on their own without any help specifically from the one who passed it to them.^13^ This ruling reconceptualizes the conscientious objection discussion by claiming that if an problematic action is going to occur anyways, then assisting with it is not as morally problematic.

Since enabling one to sin who could do so anyways without them (i.e., “same side of the river”) does not transgress the narrow definition of the biblical prohibition, a medical professional would not be required by this biblical law to object to participating in any procedure that they may regard as immoral, as long as the patient would still be able to receive it even without their involvement. However, the discussion doesn’t end there, because rabbis of the Talmud nevertheless prohibit that action under a category known as “*mesaye’a*” or “assisting” the sin of another.^14^ This thus becomes the primary focus of many of those who analyze conscientious objection from a Jewish legal perspective: what actions are prohibited because they assist another person in engaging in a transgression of Jewish law and which may one assist others with, even if they would not be comfortable with it for themselves? However, rabbinic authorities can maneuver this question with much flexibility since it is categorized as a rabbinic prohibition and not a biblical one, which lowers the degree of prohibition and allows numerous other leniencies to be considered, rendering such “assistance” permissible in certain circumstances.

For example, within this rabbinic prohibition against “assisting,” the rabbis permit assisting the person who is assisting the transgressor, since the Torah only forbids placing a stumbling block before the blind, but not placing it before another person who will then place it before the blind.^15^ This principle thus allows involvement in indirect actions. In these situations, where one can transgress without the assistance of another, many argue that the prohibition only applies if assistance is directly requested and provided at the precise moment of the transgression, but not if it takes place prior to the forbidden action.^16^ This is a very relevant ruling when it comes to behaviors such as providing referrals or information. Some are also lenient if a prohibition is only violated by inaction, such as passively allowing prohibited actions to take place.^17^ So too, if one’s livelihood depends on their professional duties, the rabbis do not require sacrificing that in order not to assist with a transgression that will be done anyways without them.^18^

The permissive category within these laws that may be the most relevant for medical professionals focuses on the claim that the only reason the rabbis prohibited one from assisting another person’s violation of Jewish law, even when that person can transgress on their own, is because they wanted people to discourage others from sinning. However, if the person who is sinning knows exactly what they are doing and has decided to do the forbidden act without concern for any Torah prohibition that might exist, the rabbis did not require one to try to prevent them from doing so, and therefore, this rabbinic prohibition against assisting them doesn’t apply.^19^ This is especially true if allowing a person to engage in a prohibited act might prevent that person from violating an even worse prohibition instead. Similarly, if one’s assistance allows others to violate a lesser prohibition, one is then required to consider the long-term consequences and favor actions that will lead to less overall sin.^20^ This likely applies to most situations of conscientious objection today, when patients turn to medical professionals for a desired intervention, but not their moral perspective on the matter.

While not all contemporary rabbinic authorities accept this approach,^21^ many do,^22^ and its ramifications for the conscientious objection discussion are profound. Although these principles arise specifically out of Jewish law, perhaps this “two sides of the river” paradigm discussed above provides a different way to look at religious objection and can be incorporated by many healthcare professionals. Following this approach would mean that a medical professional could, or perhaps *should*, be involved in most aspects of patient care for a patient who has decided to engage in an action that the medical professional would otherwise object to, since if one professional does not do it, there are others who will. Jewish law thus permits a medical professional to be involved in any service or procedure that the patient will be able to receive anyway, without their participation, if their assistance is indirect and does not directly violate Jewish law at the moment any transgression occurs.

Framed this way, one need not see this as a compromise of their values, but rather a recognition of the fact that people have options. Not only isn’t voicing conscientious objections always effective, but it could have counterproductive consequences and lead to more sinning than less. For example, research related to physician aid-in-dying has shown that paradoxically, a nonjudgmental, supportive approach from clergy has been more effective in allowing patients to consider alternatives to aid-in-dying, and even to ultimately change their minds, than active opposition to the patient’s decision (Carlson et al. 2005; Ganzini and Dobscha 2003). Studies have also shown that when clinicians take the time to listen compassionately to their patients and explore the reason(s) for their requests, that act of simply listening to the patients’ concerns helps to mitigate many of them (Matthews et al. 1993). Similarly, regarding abortion, many women who don’t have access to legal services end up attempting to do it themselves in very dangerous and often fatal ways (Rosenbaum 2019; Grimes et al. 2006).

Therefore, as long as the medical professional is not directly performing the prohibited intervention him or herself, Jewish law permits a medical professional’s involvement with what could be considered “immoral” interventions, such as:

- Writing prescriptions.^23^
- Sharing information/describing various options with a patient or making direct and effective referrals to other providers.^24^
- Serving on a board that permits abortions or other such procedures.^25^
- Writing DNR orders.^26^
- Hospice enrollment and care.^27^
- Administering anesthesia or other types of supportive care for abortion or other controversial procedures.^28^

## Conclusion

I have outlined a perspective which teaches that one does not place a stumbling block when their assistance does not directly induce more sin. Indeed, objecting can become an exercise in futility or potentially cause even worse outcomes than might ensue by refusing to participate. In particular, when the individual planning to commit an act which the professional sees as immoral will not be swayed by the protest, and the act can be carried out in any event without that individual provider, we can invoke on the “same side of the river” paradigm that arises out of Talmudic law. Rather than focusing specifically on the practitioner’s own conscience, a practitioner can then justify taking part in most interventions. In this way, one can maintain their moral integrity even without engaging in conscientious objection, while resting assured that they have not actually placed a stumbling block before the blind. It remains important to protect moral diversity, practitioner’s integrity, and allow conscientious objection in some cases, but it need not be invoked nearly as frequently as it currently is. Those who believe that it is important for religious individuals, or anyone with a strong set of moral principles, to be fully involved in health care so that they can take culturally and religiously sensitive care of patients, and have a positive impact on the way medical care is provided in our society, must find ways of working within the system. Otherwise, they might be forced to simply leave some of the most crucial interventions only to those who do not share their values.

The practice of medicine affords a profound privilege for medical professionals to have a positive impact on many lives. While medicine is a sophisticated scientific endeavor, it is also very much an art form, and every individual brings their own personality, commitments, and values to their practice. Hopefully this approach can ensure that the few instances when providers feel they must object will be taken seriously, while enabling conscientious healthcare providers to take part in most interventions, thus mitigating conflicts, allowing all patients to receive both the medical treatments and the compassion that they deserve, and our society to be served by medical professionals with both a very strong moral compass and commitment to patient-centered care.

## Footnotes

1 *Roe v. Wade*, 410 U.S. 113 (1973).

2 The legal concept of “conscientious objection” dates back at least to the 1948 Universal Declaration of Human Rights. Article 18 states, “Everyone has the right to freedom of thought, conscience, and religion…” (Pope 2010).

3 For example, medical students and residents have successfully objected to performing educational procedures on corpses without their pre-mortem consent (Fourre 2002) as well as the use of animals in medical schools (Dodge 1989), and studies have shown that many medical students have felt pressured to act unethically, which hindered their ethical growth (Hicks 2001).

4 A related argument makes the point that just as medical professionals are sometimes free to refrain from offering services based on their personal interests or professional skills, they must also be allowed to refrain based on moral or religious objections (Ancell and Sinnot-Armstrong 2017).

5 Some have argued against conscientious objection in health care by pointing to a parallel to the military context, which is that those who object to participation are required by the military to engage in some form of alternative service, and so too, they argue, should medical professionals who are unwilling to perform all of their duties (LaFollette and LaFollette 2007).

6 For these reasons, for example, in Sweden there is no right to conscientious objection. The Swedes argue that this is ethically correct, since employers have the choice of reassigning employees on a case-by-case basis, and employees can choose not to take a job if they are uncomfortable with their duties (Munthe 2017).

7 For example, a California telephone survey found that refusals to fill emergency contraception were more common in rural areas (Sampson et al. 2009). Studies have also shown that restrictions on abortion in some states render access to abortion extremely limited and sometimes insurmountable in some parts of the country (Wicclair 2019). Similarly, in all of Southern Italy, lawfully permitted abortions are not available anywhere, since so few physicians are willing to perform them (Dickens and Cook 2000). Furthermore, those who don’t have health insurance, who rely on public and/or faith-based hospitals, and who have the fewest options—often racial and ethnic minorities—are also disproportionately the most negatively impacted by conscientious objection laws.

8 This includes racial, socioeconomic, and religious discrimination. This possibility is especially problematic, since it is difficult to distinguish conscientious objection from objection from false motivations, such as cowardice or dislike (Lachman 2014).

9 *Torat Kohanim* 2:14.

10 Babylonian Talmud, Tractate *Pesachim* 22a; *Moed Katan* 17a; *Bava Metzia* 75b.

11 Babylonian Talmud, Tractate *Avodah Zarah* 6b.

12 Ibid.

13 Ibid.

14 *Tosafot, Shabbat* 3a, s.v. “*Bava*”; *Ritva* & *Ran, Avoda Zara* 6b, based on Babylonian Talmud, Tractate *Gittin* 61a.

15 Babylonian Talmud, Tractate *Avodah Zarah* 14a.

16 Mishnah, *Avodah Zarah* 4:9; Responsa *Binyan Tzion* 1:15; Responsa *Mashiv Davar* 2:32 (he adds that this would only be prohibited if one causes the action to happen sooner than it would have; otherwise it is never prohibited to assist); Responsa *Minchat Asher* 2:28, 30.

17 Responsa *Melamed L’hoil* 1:34.

18 *Ritva, Gittin* 61a s.v. “*mashelet ishah*”; Responsa *Mashiv Davar* 2:32; *Tzitz Eliezer* 19:33:9.

19 Responsa *Iggrot Moshe* YD1:72 explaining *Shach* YD 151:6 and *Dagul Merevavah* YD 151:6.

20 Ibid. R. Feinstein (quoted in previous note) argues that even those who disagree with his approach would agree in the case of permitting renting a hall to Jews who will use it for Jewishly inappropriate activities, since the alternative is for them simply to rent another hall, where they won’t serve kosher food, so it would compound their transgression of Jewish law. Furthermore, R. Shlomo Zalman Auerbach writes in Responsa *Minchat Shlomo* 1:35(1) that one may give food to a non-observant Jew who will not cover their head or recite the required blessings, since forcing them to do so could cause people to hate the Torah or religious Jews. This ruling is based on R. Akiva Eiger’s commentary on *Yoreh Deah* 181:6 that since the Torah prohibits a man from shaving his face with a razor but doesn’t prohibit a woman, it would be better for a man who is going to shave his face with a razor anyways to have a woman do it for him, even though she is thus violating the prohibition of placing a stumbling block (or only the rabbinic prohibition of “assisting” since he can do it himself). R. Akiva Eiger argues that when an action is prohibited by the Torah only due to placing a stumbling block in front of another, then it is better for a person to perform such an action, since in this way, ultimately fewer sins will be committed.

21 Responsa *Minchat Shlomo* 3:103(4); Steinberg, *Harefuah Ke’Halakhah* 2, p. 380; *Nishmat Avraham* 1:656 (pp. 820–821); See also Rosman, *Petichat Ha-Iggeret*, p. 356.

22 Responsa *Maharit* 1:97; Responsa *Minchat Asher* 2:28–30 & vol. 4 (forthcoming).

23 *Nishmat Avraham* 3:5 (p. 159 in 3rd ed).

24 Responsa *Minchat Asher*, vol. 4 (forthcoming).

25 Ibid., 2:30(6).

26 *Nishmat Avraham*, YD 339:4 (7) (510 in 3rd ed.).

27 Ibid., 339:1 (4) (499 in 3rd ed.).

28 Ibid., vol. 5 (forthcoming); *Tzitz Eliezer* 19:33(1).
